# The rationale for a multi-step therapeutic approach based on antivirals, drugs and nutrients with immunomodulatory activity in patients with coronavirus-SARS2-induced disease of different severities

**DOI:** 10.1017/S0007114520002913

**Published:** 2021-02-14

**Authors:** Sirio Fiorino, Maddalena Zippi, Claudio Gallo, Debora Sifo, Sergio Sabbatani, Roberto Manfredi, Edoardo Rasciti, Leonardo Rasciti, Enrico Giampieri, Ivan Corazza, Paolo Leandri, Dario de Biase

**Affiliations:** 1 Medicine Department, Internal Medicine Unit, Budrio Hospital Azienda USL, Budrio, 40054 Bologna, Italy; 2 Medicine Department, Internal Medicine Unit C, Maggiore Hospital Azienda USL, 40100 Bologna, Italy; 3 Gastroenterology and Hepatology Department, Unit of Gastroenterology and Digestive Endoscopy, Sandro Pertini Hospital, 00100 Rome, Italy; 4 Gastroenterology and Hepatology Department, Infective Disease Unit, Policlinico S. Orsola-Malpighi, University of Bologna, 40100 Bologna, Italy; 5 Unit of Radiodiagnostics, Ospedale degli Infermi, 48018 Faenza, AUSL Romagna, Italy; 6 Experimental, Diagnostic and Specialty Medicine Department, University of Bologna, 40100 Bologna, Italy; 7 Department of Pharmacy and Biotechnology, University of Bologna, 40100 Bologna, Italy

**Keywords:** SARS, COVID-19, Vitamins, Therapy

## Abstract

In December 2019, a novel human-infecting coronavirus, named Severe Acute Respiratory Syndrome Corona Virus 2 (SARS-CoV-2), was recognised to cause a pneumonia epidemic outbreak with different degrees of severity in Wuhan, Hubei Province in China. Since then, this epidemic has spread worldwide; in Europe, Italy has been involved. Effective preventive and therapeutic strategies are absolutely required to block this serious public health concern. Unfortunately, few studies about SARS-CoV-2 concerning its immunopathogenesis and treatment are available. On the basis of the assumption that the SARS-CoV-2 is genetically related to SARS-CoV (about 82 % of genome homology) and that its characteristics, like the modality of transmission or the type of the immune response it may stimulate, are still poorly known, a literature search was performed to identify the reports assessing these elements in patients with SARS-CoV-induced infection. Therefore, we have analysed: (1) the structure of SARS-CoV-2 and SARS-CoV; (2) the clinical signs and symptoms and pathogenic mechanisms observed during the development of acute respiratory syndrome and the cytokine release syndrome; (3) the modification of the cell microRNome and of the immune response in patients with SARS infection; and (4) the possible role of some fat-soluble compounds (such as vitamins A, D and E) in modulating directly or indirectly the replication ability of SARS-CoV-2 and host immune response.

In December 2019, a novel human-infecting coronavirus, named SARS-CoV-2 (Severe Acute Respiratory Syndrome Corona Virus 2), emerged as a very serious public health concern, causing a pneumonia epidemic outbreak in Wuhan, Hubei Province in China with different degrees of severity^(1)^. This pathological condition has been defined as ‘coronavirus disease 2019’ (abbreviated ‘COVID-19’), and the most common clinical presentation in infected subjects is represented by flu-like symptoms in 80 % of cases. About 10–15 % of infected subjects develop a more serious respiratory form. It is characterised by an interstitial pneumonia with chest discomfort, severe dyspnoea, high fever and dry cough potentially evolving into acute respiratory failure with a severe respiratory distress syndrome in about 10 % of infected subjects. The mortality rate is about 7 % of affected patients^(2)^. However, patients may also present less common symptoms, like diarrhoea, headache, myalgia or arthralgia, chills, nausea or vomiting, nasal congestion and conjunctival congestion (0·8 %)^(3)^. The epidemic has been declared a ‘public health emergency of international concern’ by the International Health Regulations Emergency Committee of the WHO^(4)^. A dramatic situation is developing in Italy with a progressively increasing number of infected subjects, mainly rather old individuals. According to current data, about 15 % of patients with SARS-CoV-2 infection develop severe forms of pneumonia, radiological signs of interstitial involvement at the computerised axial tomography. These subjects require intensive care and they are at high risk of death. The need for intensive care beds also is progressively increasing, and this condition might lead to the collapse of the Italian Health System in a very short time (data from Ministero della Salute Italiano, http://www.salute.gov.it/portale/nuovocoronavirus/homeNuovoCoronavirus.jsp?lingua=english). Unfortunately, to date, neither a vaccine nor specific proved effective treatments against this virus are available worldwide. Therefore, new therapeutic strategies are strongly required to efficaciously counteract SARS-CoV-2 as soon as possible and to establish effective antiviral approaches. Unfortunately, it must be considered that this virus has been isolated only recently, and a few articles describing its structure and genome organisation have been published. To date, studies concerning immune response against SARS-CoV-2 and the alterations induced in cell structure by this pathogen have not been studied and are not well known yet^(5)^.

## Immunopathogenesis of Severe Acute Respiratory Syndrome Corona Virus 2 infection

In the last weeks, bioinformatics analysis has been carried out on a virus genome from a patient with SARS2019-nCoV infection to compare it with other related coronavirus genomes^(6)^. According to the results, the genome of SARS2019-nCoV (now known as SARS-CoV-2) presents around 89 % nucleotide identity with the bat SARS-like-CoVZXC21 viral genome and about 82 % with that of human SARS-CoV. A wide range of viruses and host factors mutually modulate their interaction, influence the antiviral immune response and contribute to determine the pathogenesis of SARS-CoV-2^(7)^. Therefore, on the basis of the assumption that the SARS-CoV-2 is genetically related to SARS-CoV, but that its characteristics are still poorly known, we have performed a literature search to identify the reports assessing these elements in patients with SARS-CoV-induced infection, a better-defined pathologic condition since several years ago. The SARS-CoV-mediated disease resembles the SARS-CoV-2 one, and then the SARS-CoV may be helpful to better understand COVID-19. As happened for the other ‘CoV severe acute lung injury’ (such as SARS-CoV or MERS-CoV)^(8,9)^, it has been hypothesised that an imbalance in the host immune response against the CoV-2 virus may cause either the severe distress respiratory syndrome or lead to an unfavourable outcome^(10–12)^.

The aim of this paper is to examine the possible aspects of the complex loop which can develop between host and SARS-CoV-2 in brief as well as the factors and mechanisms involved in this intricate process as well as the possible immunoregulatory role of some compounds in this life-threatening condition. According to a schematic representation, some distinct phases may be recognised during the clinical course of SARS. In the first one, a robust virus replication is detectable in these patients, and it is often characterised by the appearance of fever, sore throat and non-productive cough. These symptoms generally subside in a few days with illness resolution. Nevertheless, in some individuals, a second clinical phase develops. It is characterised by elevated fever, hypoxaemia and progression to pneumonia. This step is associated with an exuberant host inflammatory response and with the sharp and vigorous decrease in virus titers^(13)^. Following this phase, about 20 % of patients develop an ‘Acute Respiratory Distress Syndrome’ with a possible fatal outcome. Lung specimens obtained from patients who have died because of SARS show several histologic tissue modifications. In particular, the most frequent alterations are represented by extensive cellular infiltrates in the interstitium and alveoli, diffuse alveolar damage with alveolar haemorrhage/oedema, hyaline membrane formation, fibrin exudation, epithelial necrosis with thickening of alveolar septa in the earlier phases and the progression to fibrosis in septa and alveoli in later stages. In particular, diffuse alveolar damage represents a critical and prominent histological feature detectable in the lungs from individuals, who have developed a fatal SARS-CoV-induced infection^(14)^. Furthermore, SARS-CoV genome and antigens have been observed in airway and alveolar epithelial cells, vascular endothelial cells, neutrophils, macrophages, monocytes and lymphocytes in samples from humans as well as from animal models^(14,15)^.

## Severe Acute Respiratory Syndrome Corona Virus 2 genome organisation and viral proteins

**Fig. 1.**
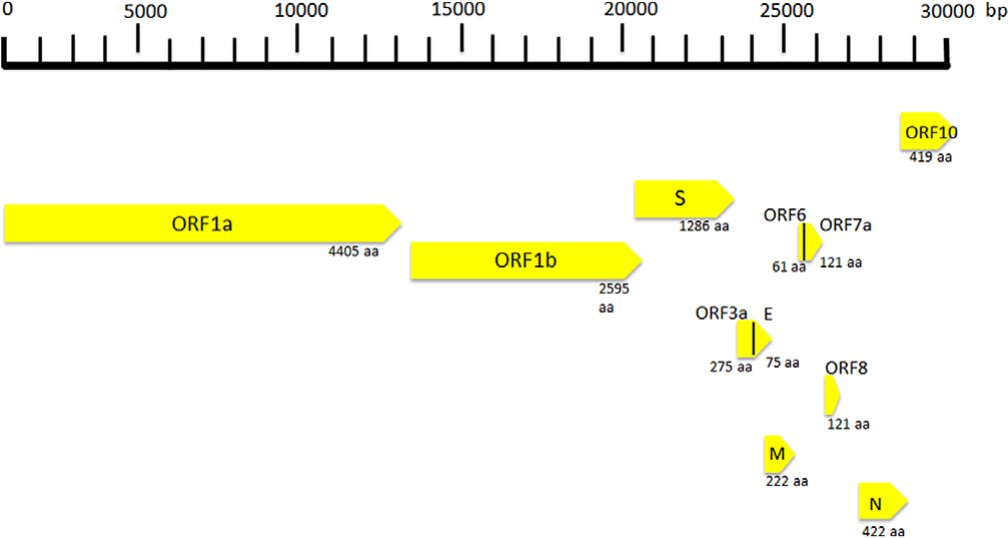
Coronavirus genome and its structural and non-structural proteins. ORF, open reading frame; aa, amino acids; N, nucleocapsid protein; S, spike protein; M, matrix protein.

SARS Cov-2 is a spherical-shaped enveloped virus, approximately 120 nm in diameter^(16)^, with the envelope consisting of a lipid bilayer derived from the host cell membrane and with spike proteins, protruding from the virion surface. These projections confer to the viral particles a crown-like morphology under electronic microscopy, so that the virus is also known as coronavirus. Each virion is composed of a positive 5’-capped and 3’-polyadenylated single-stranded RNA. The viral genome sequence is approximately 30 000 bases in length (Fig. 1)^(17)^, and it encodes several proteins including:

(1) Structural proteins, such as the nucleocapsid (N) protein, the matrix (M) protein, the small envelope (E) protein and the spike (S) glycoprotein. These proteins are in the 3’-terminus of the genome. The N protein (about 419 aminoacids in length) is detectable in the core of the viral particle and interacts with the viral RNA, generating a helical ribonucleocapsid. The N protein also binds M and nsp3 proteins, and it contributes to genome protection, viral RNA replication, virion assembly, nucleocapsid formation, generation of the mature virions and immune evasion, the E protein is a small membrane protein (about 75 amino acids in length), regulating viral particle assembly, budding and pathogenesis. It binds M, N, 3a and 7a proteins. The M protein is a membrane/matrix protein (around 222 amino acids in length), and it is involved in viral particles assembly and budding via the recruitment of other structural proteins. In particular, the M protein interacts with N protein for RNA packaging into virion and with some accessory proteins like 3a and 7a. The S, E and M proteins together create the viral envelope. The spike protein is synthesised as a precursor (around 1273 amino acids in length) and then it is cleaved into glycosylated subunits, S1 and S2. During virus infection, S1 allows the attachment of Sars-CoV-2 to a specific receptor of host’s cell, known as angiotensin-converting enzyme 2 receptor, while S2 mediates the fusion between virus and cell membrane.
(2) Non-structural proteins (NSP), to date, sixteen NSP have been described, but, for most of them, the function is not yet known. Among the better characterised proteins there are:

(a) NSP1 (about 180 amino acids), it modulates viral gene expression and immunoevasion by influencing interferon-mediated signalling, (b) NSP2 (about 638 amino acids), it perturbs intracellular microenvironment and alters intracellular signalling paths, (c) NSP4 (with NSP3) is needed for the assembly of the virion particles during the course of viral replication, (d) NSP7 and NSP8, they act as a cofactor for the RNA-dependent RNA polymerase (known as NSP12) activity with NSP9. These proteins regulate viral replication, (e) NSP12 (about 932 amino acids) represents the RNA-dependent RNA polymerase, and it is involved both in replication and in transcription of the SARS-CoV-2 genome and (f) NSP13, NSP14 and NSP15, they modulate viral replication.

Open reading frames (ORF), a variable number of (6–10) ORF have been described. The first two ORF at 5′ untranslated region code for polyprotein (ORF1a and ORF1b). The ORF1a produces a polypeptide 1a that is cleaved into 11 NSP and ORF-1b produces the polypeptide 1b that it is cleaved into fifteen proteins. The viral proteases NSP3 and NSP5 are involved in this process. These NSP are required for virus replication. Further, eight accessory proteins designated ORF-3a, 3b, 6, 7a, 7b, 8a, 8b and 9b have been described in the viral genome. These sequences are interposed among the structural genes (Fig. 1)^(18)^ and exert different and complex viral functions. Among the most important functions of the accessory proteins, ORF-3a binds to proteins 7a, M, S and E and activates cell inflammatory mediators and contributes to the generation of cytokine storm. ORF-6 acts as antagonist of type I interferons (IFN) and promotes viral escape from the host innate immune system. Following the entry into cells, the genomic RNA is translated to generate NSP from the ORF1a and ORF1b. The viral genome serves also as the template for replication and transcription, via the activation of NSP12, which exerts RNA dependent RNA polymerase activity. Furthermore, also negative-sense RNA intermediates are synthesised and function as templates for the generation of positive-sense genomic RNA (gRNA) and subgenomic RNA (sgRNA). The gRNA and the structural proteins are assembled and generate the viral progeny. The sgRNA serve as template for the structural proteins (spike-, envelope-, membrane- and nucleocapsid proteins) and accessory proteins^(19)^.

## Defective and dysfunctional immune response in patients with Severe Acute Respiratory Syndrome Corona Virus 2-related infection

A comprehensive theory of the pathogenesis for SARS-CoV-2 infectious disease is still lacking, but it has been proposed for SARS-CoV in the past^(14)^, and some preliminary studies about SARS-CoV-2 have been published or are in progress^(20)^.

**Fig. 2.**
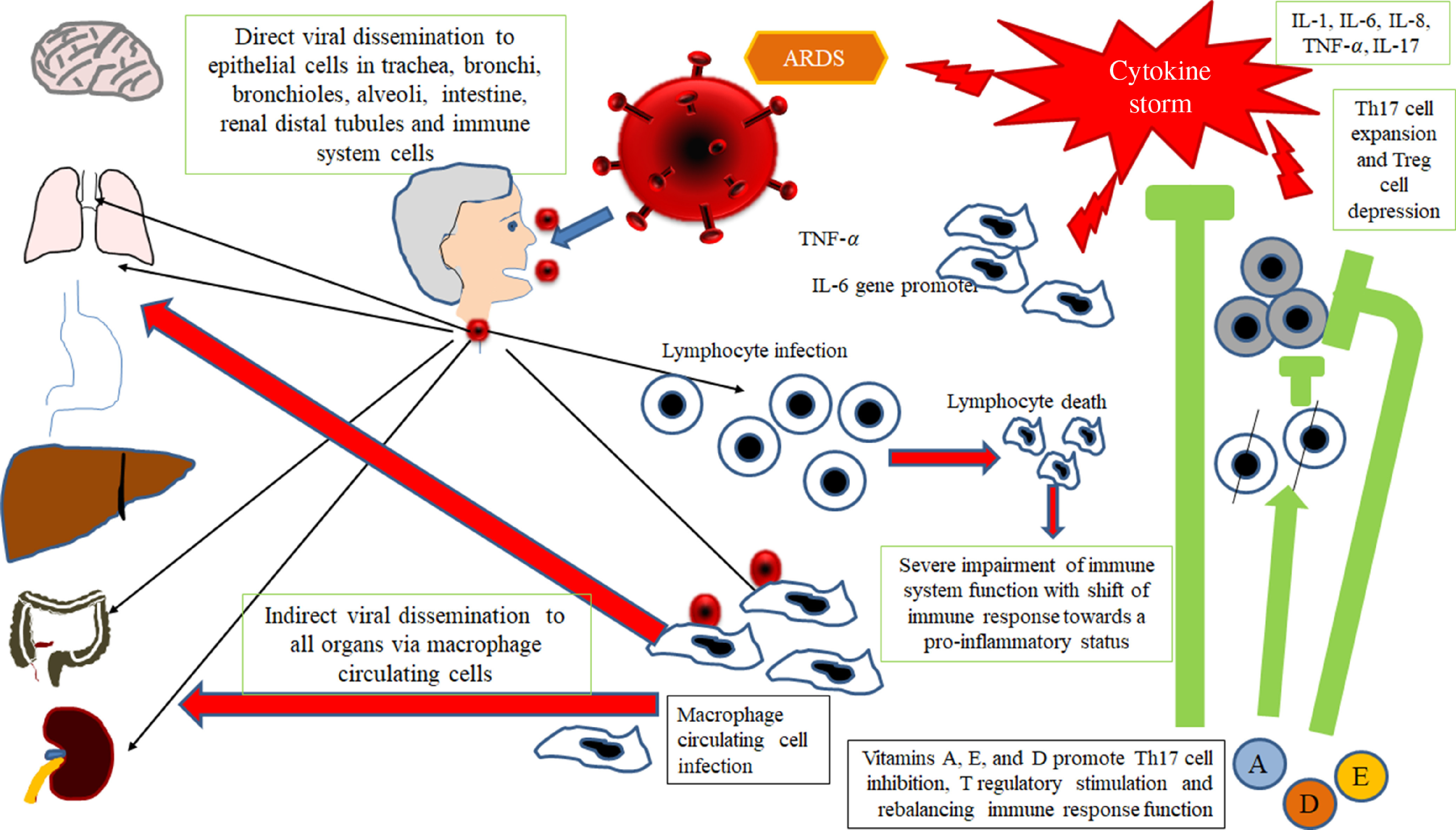
Gu’s hypothesis, concerning SARS-CoV infection^(14)^. A similar scheme may be considered with the purpose to explain the pathogenesis of SARS-CoV-2. The SARS-CoV infects the human body through the respiratory tract, entering the epithelial cells of the trachea, bronchi, bronchioles and lungs. In this context, the virus also colonises resident, infiltrating and circulating immune cells. Then, the virus disseminates to all human organs, being carried by the infected circulating immune cells and spread to different types of cells in other organs. The immune cells of the spleen, peripheral and central lymph nodes, other lymphoid tissues are colonised and damaged by the virus. Furthermore, the mucosa of the intestine, the epithelium of the renal distal tubules, the neurons of the brain and the macrophages in different organs are also involved. According to this hypothesis, it may be assumed that infected circulating immune cells spread to the mucosa-associated lymphoid tissue (MALT) and bronchus-associated lymphoid tissue (BALT) The immune defence is significantly impaired and infected patients may develop pneumonia with different degrees of severity and experiment a rapid deterioration of clinical conditions. Aged subjects with chronic diseases have often a compromised immune function, generally develop more severe clinical pictures and present a more elevated mortality in comparison with healthy subjects. The severity of the immune cell damage more than the extent of the lesions detectable in the lungs suggests the patient’s immune status, and his lymphocyte count probably represents the main predictor of his clinical evolution. Viral load also may exert a crucial impact on the strength and efficacy of the patient’s immune response. The possible action of fat-soluble vitamins in improving immune response activity is indicated. ARDS, acute respiratory distress syndrome.

Therefore, taking into account all available data in SARS-CoV infection and considering SARS-CoV-2 as a virus with similar characteristics and immunopathogenic effects to SARS-Co-V, it may be hypothesised that the deleterious events in patients with the most severe forms of the COVID-19 are the results both of an excessive or inadequate immune response of the host^(14,21,22)^. According to Gu’s hypothesis, the SARS-CoV infects the human body through the respiratory tract, entering the epithelial cells of the trachea, bronchi, bronchioles and lungs^(14)^ (Fig. 2). In this context, the virus colonises also resident, infiltrating and circulating immune cells. Then, the virus disseminates to all human organs, being carried by the infected circulating immune cells and spread to different types of cells in other organs. The immune cells of the spleen, peripheral and central lymph nodes, other lymphoid tissues are colonised and damaged by the virus. Furthermore, the mucosa of the intestine, the epithelium of the renal distal tubules, the neurons of the brain and macrophages in different organs are also involved. According to this hypothesis, it may be assumed that infected circulating immune cells spread to the mucosa-associated lymphoid tissue, bronchus-associated lymphoid tissue and nasopharynx-associated lymphoid tissue. No data are available concerning the possible virus-mediated alterations in the function of these lymphoid compartments in patients with SARS-CoV-2 infection. The immune defence is significantly impaired and infected patients may develop pneumonia with different degrees of severity and experiment a rapid deterioration of clinical conditions. In particular, aged subjects with chronic diseases have often a compromised immune function, generally develop more severe clinical pictures and present a more elevated mortality in comparison with healthy subjects^(23)^. According to Gu’s study, the severity of the immune cell damage more than the extent of the lesions detectable in the lungs suggests that the patient’s immune status and his lymphocyte count probably represent the main predictor of his clinical evolution^(14)^. Viral load also may exert a crucial impact on the strength and efficacy of the patient’s immune response^(23)^. During the course of SARS-CoV and CoV2 diseases, an activation of the immune response progressively develops, leading to a self-maintaining and self-increasing inflammatory state. High serum levels of pro-inflammatory cytokines (IFN-*γ*, IL-1, IL-6, IL-12 and TGFβ)^(24,25)^ and chemokines (CCL2, CXCL10, CXCL9 and IL-8) have been detected in SARS patients, who develop the most severe clinical forms of disease in comparison with subjects with a milder illness^(26–28)^. Furthermore, a strong pro-inflammatory Th1 and Th17 response has been observed in patients with MERS-CoV (Middle East respiratory syndrome Coronavirus) infection, with increased concentrations of IFN-*γ*, TNF-*α*, IL-15 and IL-17^(29)^. In humans, Th17 cells (T-helper 17) can be induced by IL-6 and IL-1*β*^(30)^. Experimental research in *in vitro* models of cultured cells has examined the pattern of SARS-CoV proteins and has allowed to identify the potential pro-inflammatory role of some among them in the pathogenesis of SARS. In particular, nucleocapsid (N) and spike (S) SARS-CoV proteins possess direct binding sites on several specific DNA sequences, localised in the promoter region of a wide series of interleukins and cytokines^(31,32)^.

It may be hypothesised that SARS-CoV-2-induced disease with severe clinical courses and with a fatal outcome is characterised by a massive release of a wide spectrum of cytokines, leading to the cytokine release syndrome (CRS)^(33)^. A more detailed discussion of this topic is beyond the scope of this work, and it will be the subject of a further paper. Therefore, on the basis of these concepts and observations, a proper modulation or control of the exuberant inflammatory response, developing in the course of SARS-CoV-2 infection, might be a key strategy for the treatment of the patients with severe forms of SARS-CoV-2 infections and, probably, it might also prevent the evolution of the illness towards an unfavourable outcome.

## Factors involved in the inflammatory immune response in patients with Severe Acute Respiratory Syndrome Corona Virus 2

Multiple factors may contribute to explain the exuberant inflammatory response, detectable in this severe disease and should be considered in the strategy of treatment. Overall, these elements may contribute to determine the differences in clinical course and severity of illness in patients with COVID-19. The following points should be considered:

(i) Rapidity of viral replication and load of viral proteins, mainly proteins causing the release of IL-1, IL-6, IL-8 and TNF-*α*;
(ii) Anatomical human compartment or organ predominantly infected by the virus;
(iii) Cytokine storm and antiviral impaired immune response.

## Possible role of some drugs and nutrients in modulating directly or indirectly the replication ability of Severe Acute Respiratory Syndrome Corona Virus 2 and host immune response

**Fig. 3.**
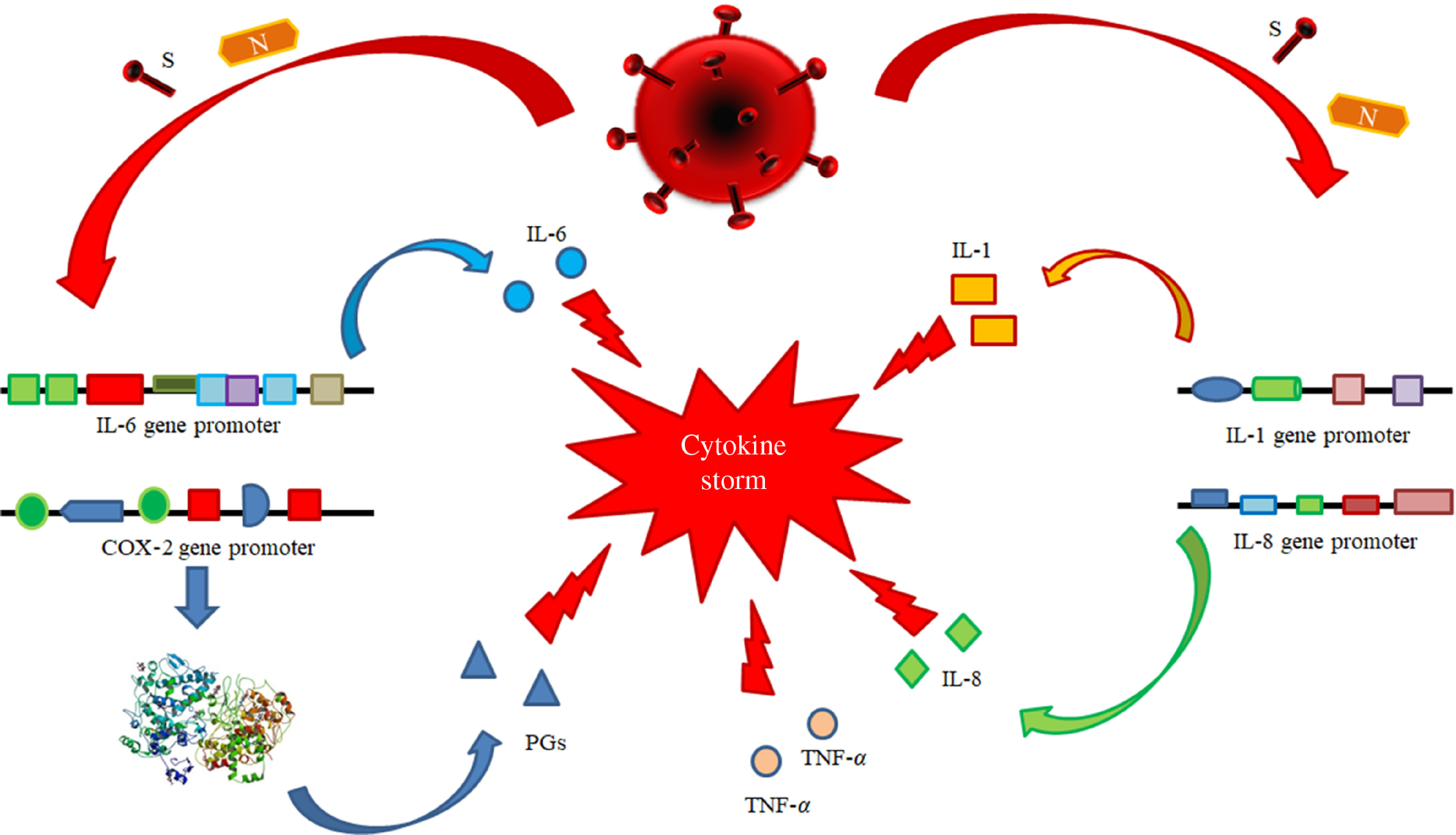
Pathogenetic mechanisms involved in the cytokine storm syndrome. N and S viral proteins possess some target sequences on the DNA in the nucleus of human cells. Some binding motifs are detectable in the promoter of some cell genes, encoding key cytokines or enzymes involved in inflammatory process, such as IL-1, IL-6, IL-8, TNF-*α* and cyclo-oxygenase (COX)-2. Subjects with an immune system dysregulation (e.g. aged individuals with chronic diseases and impaired immune system function) are particularly at risk to develop this life-threatening condition.

**Fig. 4.**
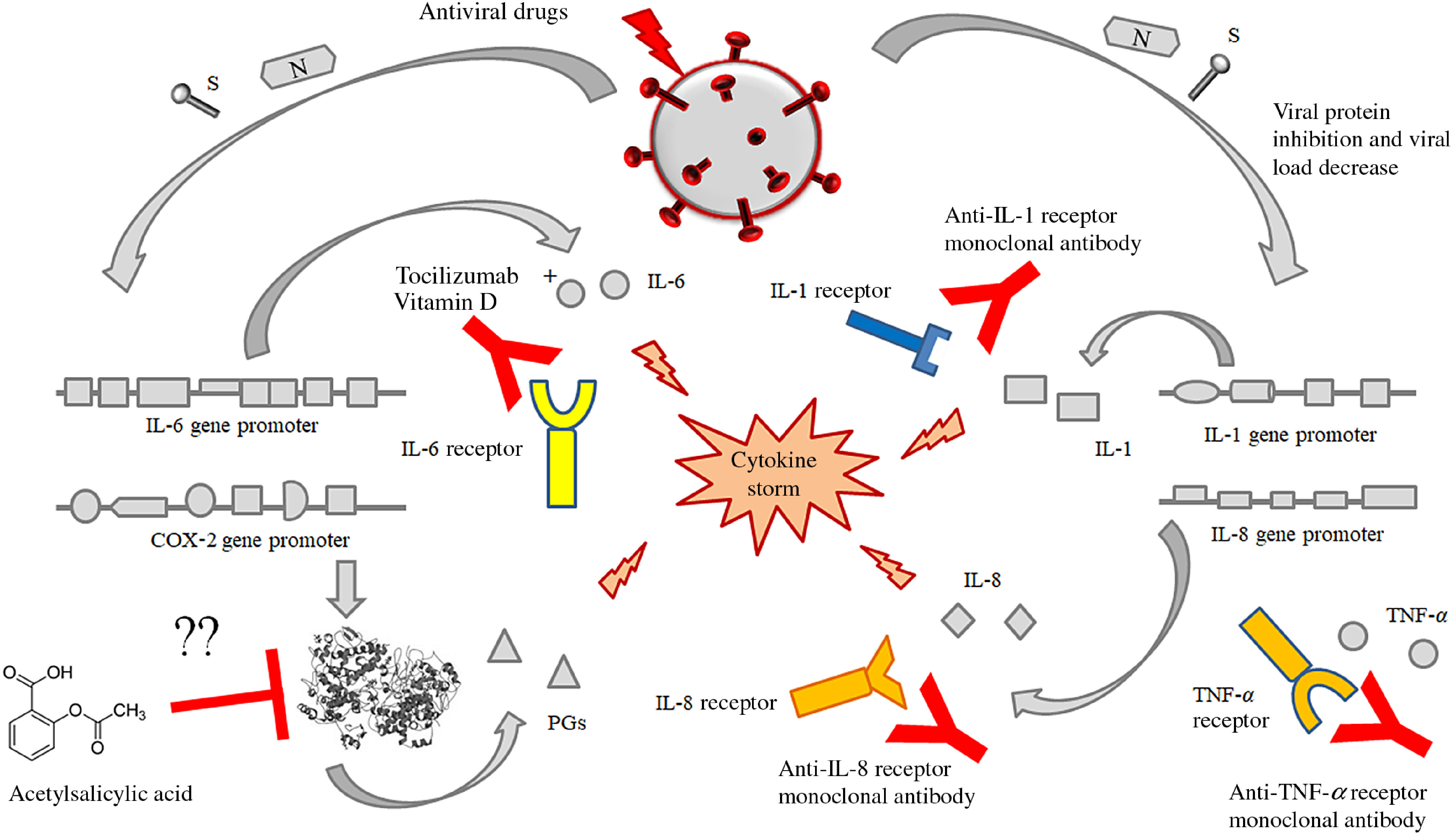
Possible or putative therapeutic targets potentially useful for the prevention or treatment of the cytokine release syndrome (CRS) by means of acetylsalicylic acid (although perplexity has been expressed about this treatment), monoclonal antibodies against the receptors of some interleukins like IL-6, IL-1 alone or in association with some fat-soluble vitamins (mainly vitamin D). This figure provides the conceptual hypothesis that multiple therapeutic targets may be considered. To date, there are no certainties on the efficacy of any therapies, alone or in combination, which may have some efficacy in the treatment of the CRS in patients with SARS-CoV-2 infection.

On the basis of all these immunopathogenic and clinical observations and considerations, a potential useful therapeutic rescue strategy for the treatment of patients affected by severe forms of SARS-CoV-2 infection could include the following points:

(i) Antiviral therapy with the currently available drugs, which have been demonstrated to be effective in reducing or in inhibiting replication of RNA-viruses (HCV, HIV and Ebola virus) in previous trials or of SARS-CoV-2 itself in very preliminary reports and anecdotal cases. This therapy should be administered as soon as possible to counteract SARS-CoV-2 replication with the main purpose to decrease the synthesis and the release of some crucial viral proteins (nucleocapsid and spike proteins) detectable in the cytoplasm and in the nucleus of the infected cells. The inhibition in the synthesis of these proteins should promote the decrease of their amounts and remove the persisting stimulus, which induce the transcription and the translation of the pro-inflammatory cytokines. This strategy may prevent the persistence of the self-maintaining and self-stimulating pro-inflammatory loop in the body tissues of infected individuals, mainly in the lung, associated with the release of the pro-inflammatory cytokines. The result of this therapy is the inhibition of the so called ‘cytokine storm’ and the block of its related deleterious effects (Figs. 3 and 4). A high viral replication in infected cells may be associated with the release of elevated N and S protein amounts. The binding to the promoters of the pro-inflammatory cytokines and enzymes may induce a hyper activation in the transduction and translation of these genes. As consequence, elevated amounts of pro-inflammatory cytokines are synthesised and secreted. The massive release of these mediators is associated with the development of the CRS. Subjects with an immune system dysregulation (e.g. aged individuals with chronic diseases and impaired immune system function) are particularly at risk to develop this life-threatening condition.

To date, some drugs have demonstrated potential efficacy in the treatment of SARS-CoV-2-infected individuals, including (a) approved nucleoside analogues (Favipiravir and Ribavirin) and experimental nucleoside analogues (Remdesivir and Galidesivir) able to inhibit the RNA-dependent RNA polymerase and to block viral RNA synthesis in a broad spectrum of RNA viruses, including human coronaviruses^(34)^; (b) approved protease inhibitors including disulfiram, lopinavir, indinavir, saquinavir, ritonavir, atazanavir and darunavir have been shown to have activity against SARS-CoV-2^(35)^.

(ii) Immunomodulatory therapy, including (a) monoclonal antibodies against IL-6 (as suggested in preliminary reports) and eventually against IL-1 and/or IL-8 as well as against cyclo-oxygenase (COX) inhibitors, like aspirin or other non-steroidal anti-inflammatory drugs with the purpose to stop or to prevent the strong inflammatory response and the release of further cytokines and mediators of inflammation.

Very preliminary observation suggests that the block of IL-6 pathway cascade may have a beneficial effect in patients with severe forms of SARS. Tocilizumab is a humanised anti-IL-6 receptor subunit *α* (anti-IL-6 R) monoclonal antibody approved in numerous countries throughout the world, for the treatment of rheumatoid arthritis, with moderate to severe active rheumatoid arthritis, refractory to methotrexate^(36)^. In patients with rheumatoid arthritis, the inhibition of IL-6 leads to Th1 and Th17 suppression and Th2 expansion via activation of T-regulatory (T-reg) cells^(37,38)^.

It is conceivable that the observed improvement in clinical conditions of patients suffering from severe forms of SARS-CoV-2 infections depends on the attenuation of the CRS. Well-designed clinical trials are need in a very short time to test the efficacy and the safety of this potentially very promising therapeutic approach (unpublished observations). No data are available on the possible efficacy and safety of acetylsalicylic acid as well as the duration for an effective treatment. To date, the use of aspirin as an option for the treatment of acute respiratory distress syndrome, with the purpose to inhibit COX-2 activity, has been proposed^(39)^. Inhibition of COX-2 might attenuate the CRS, but only one experimental study in animals has tested a possible role of aspirin in acute lung injury. Aspirin has been reported to protect mice in a two-event model of transfusion-related acute lung injury^(40)^. The lack of studies on this topic makes it difficult to hypothesise the role of aspirin in the treatment of these patients and requires further studies.

Other possible, but, to date, not tested anti-SARS-CoV-2 compounds with potential usefulness against virus or against its related complications may be represented by some fat-soluble vitamins. Therapeutic regimens with fat-soluble vitamins’ administration (such as A, D and E) are based on their immunoregulatory activity, due to their ability to exert a protective role for the maintenance of a proper function of the immune response as well as on their antioxidant activities with potential beneficial effects in attenuating the oxidative stress, which emerges in cells and tissue, during both acute and persistent viral infections^(41)^. Oxidative stress represents one of the first events developing as defence mechanism, when a pathogen (bacteria, fungi or viruses) infects a host. In normal conditions, host’s cells in general and immune cells in particular produce reactive species, including reactive oxygen species and reactive nitrogen species, which act as mediators both in physiological and in pathological processes. The synthesis and release of these chemical compounds by immune cells, like macrophages, neutrophils and monocyte, are increased, following an infection^(42)^. Reactive species counteract the invading pathogens, contribute to hinder them and to control the infection via regulation of cellular signalling paths, cytokines release, growth factors transcription, proliferation, gene expression, adhesion, metabolism and apoptosis. Nevertheless, these chemical specimens also display harmful actions and their hyperproduction may lead to DNA, lipids and proteins oxidation resulting in their damage and in alteration of cellular integrity and homoeostasis^(43)^. Cells possess an antioxidant defence system to prevent oxidative injury, including enzymatic (superoxide dismutase, catalase and glutathione peroxidase) and non-enzymatic components (like vitamin E), This imbalance could result from a lack of antioxidant capacity or an overabundance of oxygen reactive species. When the abundance of reactive oxygen species overcomes the host’s antioxidant capacity, an unbalance of cell oxidant–antioxidant status results. This condition is defined ‘oxidative stress’ and may induce a potential cellular and tissue damage. Since several years ago, it is well known that a wide spectrum of viruses including hepatitis B virus (HBV)^(44)^, hepatitis C (HCV)^(45)^, delta (HDV)^(46)^, herpes viruses^(47)^ and respiratory viruses^(48,49)^ may affect cellular redox balance by increasing reactive species such as superoxide and nitric oxide and inhibit the synthesis of antioxidant enzymes such as superoxide dismutase, catalase and glutathione peroxidase^(50)^. Furthermore, although the available data are still partial, some studies have shown that patients with SARS-CoV-2 infection also present an increased production of reactive species, with an alteration of host’s antioxidant system, exerting a major role in the pathogenesis, progression and severity of this pathological condition^(51,52)^.

Previous studies have shown that vitamins A, D and E possess antioxidant effectiveness counteracting peroxidation of lipids incorporated in plasma membrane cells, in membranes of mitochondria, endoplasmic reticulum and lysosomes as well as the oxidative damage of DNA and of macromolecular protein structures inside the cytoplasm^(53–58)^.

The antioxidant effects and mechanisms of vitamins A, D and E will be discussed in detail in the section entitled: ‘Potential anti-SARS-Cov-2 biological activity of the vitamins A, D and E may be associated with their molecular structure’.

The rationale for the use of these compounds with the purpose to treat SARS-CoV-2 infection deserves a conceptual explanation. Fat-soluble vitamins possess numerous cellular targets and can modulate a wide variety of cell activities at various levels^(59)^. In this paper, we will consider in brief the regulatory activities of fat-soluble vitamins on the immune system functions and on the inflammatory response. These compounds possess pleiotropic effects and may exert a systemic direct antiviral- or immunomodulatory effects.

The following points must be considered:

(i) A large series of clinical studies have shown that the serum concentrations of vitamins A, E and D are decreased in patients with some chronic viral infections, like HBV, HCV and HIV^(60,61)^, in comparison with uninfected individuals as well as in aged patients^(62)^.
(ii) The deficiency of vitamins D, E and A is associated with higher levels of viral replication as well as with higher values of inflammatory cytokines, like IL-6 and TNF-*α*^(63–65)^.

Vitamin E has been shown in several trials to enhance the immune response and resistance to infections^(66)^. All-trans retinoic acid is an active metabolite of vitamin A (VA), and it has been shown to modulate immunity. It induces the differentiation of CD4^+^ T-cells into T-reg cells but inhibits the differentiation of Th17 cells, thereby it contributes to the maintenance of the Th17/T-reg cell balance^(67)^.

Some vitamins, like vitamins E, D and A, have been used in clinical trials for the treatment of patients with persistent viral infections, including HBV, HCV and HIV. These micronutrients have been demonstrated to enhance both the innate and the adaptive immunity against these pathogens^(61,68–73)^ and to decrease susceptibility of CD4+ T lymphocytes to HIV-1 infection^(74)^. Furthermore, vitamins A, D and E have been suggested to improve innate and adaptive immune response against respiratory viruses, including influenza virus, rhinovirus and respiratory syncytial virus both *in vivo* and *in vitro* studies^(75)^. Possible antiviral role of vitamin E has been already suggested several years ago in patients with respiratory infections^(76)^, but very interesting and promising anti-HBV effects have been observed in clinical trials, involving a small number of children^(77,78)^ and adult patients^(79)^, with HBeAg-positive and HBeAg-positive/negative chronic hepatitis. The possible rationale of vitamin E use in these patients and the potential targets of direct or indirect antiviral effects mediated by vitamin E have been widely discussed in a previous systematic review^(70)^.

(iii) Fat-soluble vitamins possess well-known multiple nuclear and cytoplasmic targets in all the different types of mammalian cells, and they may modulate and regulate an elevated number of intra- and extracellular pathways via a direct binding to regulatory regions in a large series of genes critical for the maintenance of cell homoeostasis, via modulation of a wide series of cell functions^(70,80)^.

## Possible mechanisms underlying the effects of fat-soluble vitamins in counteracting Severe Acute Respiratory Syndrome Corona Virus 2 infection

On the basis of this brief revision of the reported antiviral activities of vitamins A, D and E against different human viruses (both DNA and RNA viruses), reported in *in vivo* and in *in vitro* studies, it may be hypothesised that these micronutrients may have possible beneficial effects also in counteracting SARS-CoV-2 infection. Several elements may have a role in these events, and their accurate definition and understanding may contribute to increase our knowledge of SARS-CoV-2 pathogenesis and to improve the treatment of this pathogen.

### Potential anti-Severe Acute Respiratory Syndrome Corona Virus 2 biological activity of the vitamins A, D and E may be associated with their molecular structure

Several studies have underlined that a key event in the development of a productive viral infection is represented by the optimal interaction between some components of the host cell plasma membrane and some proteins of the virus envelope^(81)^. This process allows the entry of the pathogen into the cell and affects the infective ability of each virus as well as its tissue tropism, its local or diffuse replication and dissemination and, as further aspects, its virulence and its pathogenicity^(82)^. SARS-CoV-2 infects permissive host’s cells by means of its glycoprotein S (spike protein), which interacts with the angiotensin-converting enzyme 2 receptors on human cells.

**Fig. 5.**
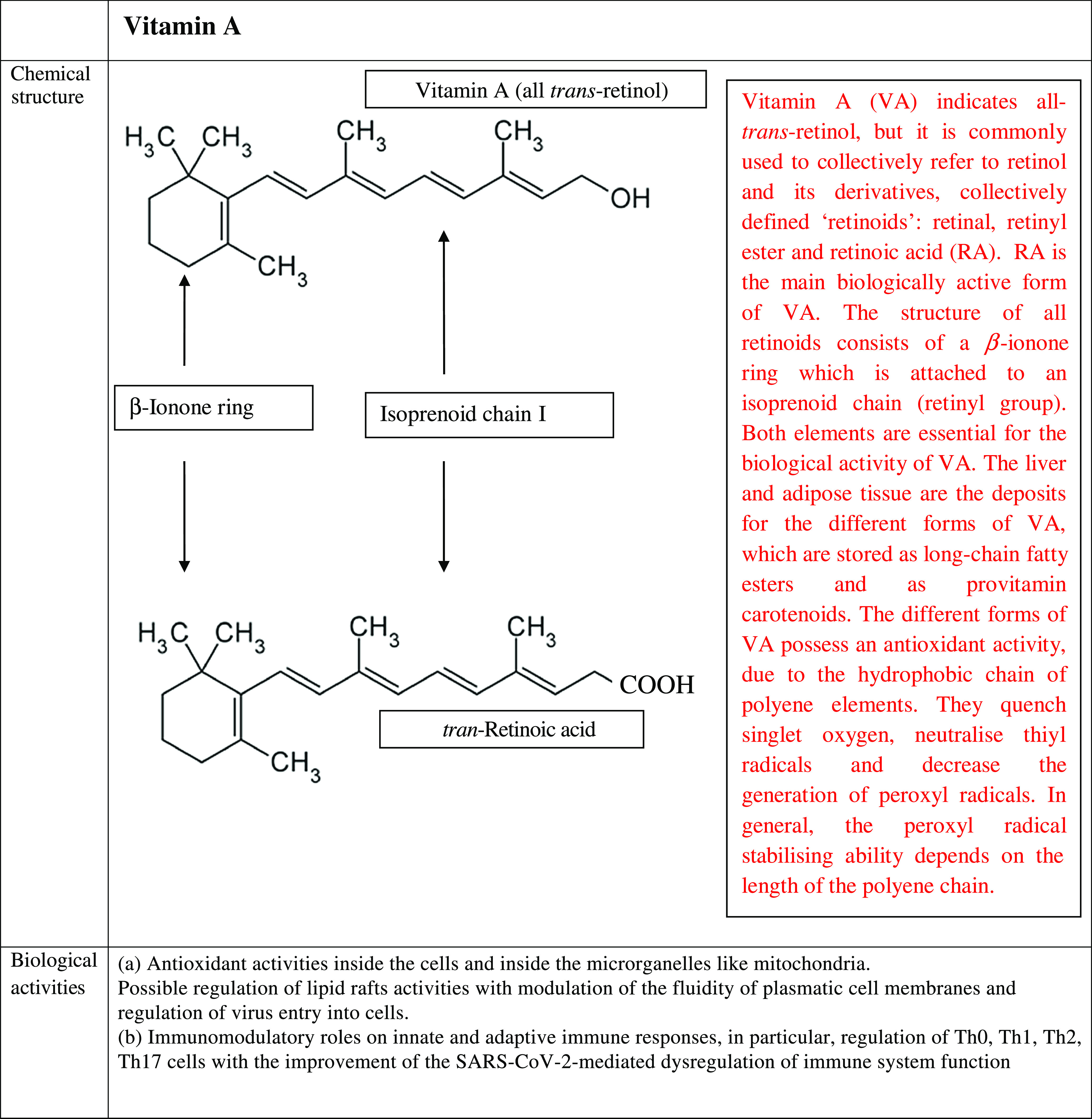
Chemical structure, biological activities and use as antiviral treatments of vitamins A, D and E. AVT, antiviral therapy; BetaC, betacarotene; C, controls; CT, controlled trial; CHB, chronic hepatitis B; CHC, chronic hepatitis C; DB, double blind; F, female; FU, follow-up; HBV, hepatitis B virus; HCV, hepatitis C virus; I, Intervention group; IU, international units; M, male; NT, not treated; PC, placebo controlled; R, randomised; RBP, retinol-binding protein; SVR, sustained virological response; T, treated; y, years; TGF, transforming growth factor; VA, vitamin A; VC, vitamin C; VD, vitamin D; VE, vitamin E.

Following the binding, spike protein divides into two subunits (S1 and S2). S1 protein includes a receptor sequence for the binding to the peptidase domain of angiotensin-converting enzyme 2, whereas S2 is involved in the process of fusion between plasma membranes and the envelope of viral particles^(83)^. Available data suggest that the lipid composition of cell plasmatic membranes may affect the entry into host’s cells of several viruses, and it may modulate their replication. In particular, some studies have shown that the entry of several viruses, including SARS-CoV-2, into the host’s cells is mediated by some specialised microdomains with specific constituents, detectable in plasmatic membrane cells^(84)^. These complexes have been defined lipid rafts, they are rich in cholesterol, sphingolipid and proteins and act as platforms that modulate the signals and the cascade pathways in cell membrane^(85)^. It has been suggested that lipid rafts facilitate the interaction between the spike protein and its ACE2 receptor and favour the entry of SARS-CoV into the cells via the fusion of the viral lipid envelope with the plasma membrane of the susceptible cells^(86)^. This event is followed by the endocytosis of virions. In particular, both cholesterol and fatty acids regulate these processes, and it has been shown that the pharmacological depletion of cholesterol activity may inhibit the attachment of several viruses, including SARS-CoV-2, to host’s membrane cells^(87)^. Furthermore, viruses themselves may modulate cell lipid metabolism and may induce a modification in the total specific lipid content of the cellular plasmatic membranes. It has been suggested that lipids in these structures may undergo an oxidative process via the activation of canonical lipase pathways. The changes in the lipid membrane composition are associated with an alteration in its fluidity and permeability. The variation of these physical parameters may have a crucial impact in the infectivity of viruses^(85)^. Taking advantage from all these studies and observations, it may be hypothesised that the biological actions of vitamins A, D and E against SARS-CoV-2 could depend on the ability of these vitamins to modulate the rigidity/fluidity of the plasmatic membrane cells. These effects may be explained by the structure of these micronutrients. Fig. 5 summarises the chemical structures of vitamins A, D and E.

VA is a term indicating retinol and its derivatives, collectively defined ‘retinoids’. They are essential nutrients for all vertebrate animal species. Two dietary sources of VA exist in nature such as preformed retinoids and provitamin A (pro-VA) carotenoids. Among carotenoids, *β*-carotene represents the most important precursor of VA. Furthermore, retinol, retinal and retinoic acid are the forms of this micronutrient detectable in the body^(88)^. All these compounds are toxic at elevated concentration; therefore, they are bound to proteins both in the intracellular and extracellular microenvironments. Retinoic acid (RA) is the main biologically active form of this micronutrient. The structure of all forms of VA consists of a *β*-ionone ring which is attached to an isoprenoid chain (retinyl group). Both elements are essential for the biological activity of these micronutrients. The liver and adipose tissue act as deposits for the different forms of VA, which are stored as long-chain fatty esters and as provitamin carotenoids. The main functions of the biological active forms of these micronutrients include vision, immunity, cell differentiation, embryological development, cellular differentiation and proliferation as well as antioxidant activity^(89)^. The different forms of VA possess an antioxidant activity, due to the hydrophobic chain of polyene elements. They can quench singlet oxygen, neutralise thiyl radicals and decrease the generation of peroxyl radicals. In general, the peroxyl radical stabilising ability depends on the length of the polyene chain, the longer it is, the greater is the peroxyl radical stabilising activity. Furthermore, when O_2_ tension increases, the different biological forms of VA can autoxidise, and this function depends on their structures. This activity is observed in human tissues, where low oxygen tensions exist physiologically. Therefore, retinoids are very effective antioxidants in this condition^(90)^. VA promotes the maintenance of levels and structure of tight junctions among the cells in the small intestine. Diets with restriction in VA in animal models cause an impairment in the architecture and tight junctions barrier in the cells of the small intestine. This damage involves villi and it is characterised by a decrease in amount of tight junction proteins, such as Zonula Occludens-1, occludin and claudin-1^(91)^. It is well known that retinoic acid modulates the expression of several cellular gene programmes via the activation of the nuclear RA receptors (RAR). They are represented by three subtypes (RAR*α*, RAR*β* and RAR*γ*). These elements are ligand-inducible transcriptional regulators and heterodimerise with retinoid X receptors (RXR). RAR possess a domain for the binding to nuclear DNA. Interestingly, a fraction of RAR*α* is in lipid rafts. In these specialised structures, there are some signal-transducing molecules, like protein kinase. To date, it is not known whether the binding of RA to RAR *α* may induce modification in fluidity of plasma cell membranes and whether this event may influence viral infectivity. Further studies are needed to clarify this point^(92)^.

The term vitamin D indicates a spectrum of fat-soluble micronutrients with multiple biological effects. In humans, the most important members of this group are represented by vitamin D_2_ (VD_2_) (ergocalciferol) and by vitamin D_3_ (VD_3_) (cholecalciferol)^(93)^. Vitamin D_3_ is the most relevant form of vitamin D. It is synthesised from 7-dehydrocholesterol through a chemical reaction that is dependent on sun exposure (specifically UVB radiation). During this process, the B ring of this chemical compound opens and becomes a less rigid structure. This event occurs in the lipid bilayer of the plasma membranes inside the cells, which are localised in the lower layers of skin epidermis. Alternatively, vitamin D_3_ can be acquired with the diet. Vitamin D_3_, which is introduced with the diet or is synthesised in the skin, is biologically inactive. It undergoes two enzymatic hydroxylation steps, the first occurs in the liver and the second in the kidneys. In particular, cholecalciferol is turned into calcifediol (25-hydroxycholecalciferol) and ergocalciferol into 25-hydroxyergocalciferol in the liver. Calcifediol is converted into calcitriol, known as 1,25-dihydroxycholecalciferol, via a further hydroxylation in the kidneys^(93)^. This is the biologically active form of vitamin D. Calcitriol has a major role in regulating the concentration of Ca and P, and it is involved in remodelling of bone. Furthermore, it also has other effects, including some on cell growth, neuromuscular and immune functions, and down-regulation of inflammation. Geometry of the rings A and C and side chain in its structure can affect some biological activities of vitamin D_3_, like its differentiative and antiproliferative abilities as well as its resistance to catabolism. Since several years ago, vitamin D_3_ has been shown to possesses *in vitro* and *in vivo* antioxidant properties. In particular, vitamin D_3_ acts as a membrane antioxidant with inhibitory activity on iron-induced lipid peroxidation of brain liposomes membrane^(55)^, or it has been able to suppress the process of lipid peroxidation in rats with deficiency in vitamin D_3_^(94)^. Furthermore, this micronutrient has been reported to reduce OS by up-regulating antioxidative defence systems, including glutathione content, glutathione peroxidase and superoxide dismutase in cultured astrocytes and in hepatic cells^(53)^. Furthermore, vitamin D promotes the maintenance of tight junctions, gap junctions and adherens junctions in the cells (e.g. by E-cadherin)^(95,96)^. 1,25-Dihydroxycholecalciferol is not detectable inside the lipid bilayer in cellular plasma membranes, but it exerts its modulatory activities by stimulation of two receptors: a nuclear vitamin D receptor and a membrane receptor ERp60. Vitamin D_3_ binding to these receptors induces the activation of several cytoplasmic pathways, including the activation of several protein kinases. Both receptors are incorporated into the lipid rafts in plasma membrane cells, and this evidence suggests the hypothesis that these microdomains have a major role in the mechanism of 1*α*,25(OH)_2_D_3_ action. It is conceivable that vitamin D_3_, by binding to its cognate receptors, may modulate the rigidity/fluidity of membrane cells and may modulate viral infectivity^(97)^. The term vitamin E indicates a series of related compounds, each of these is composed by a 6-chromanol ring and by a polyisopentenyl side chain^(98)^. This chain is either saturated (tocopherols) or unsaturated with three double bonds, detectable at positions 3’, 7’ and 11’ (tocotrienols). Tocopherols and tocotrienols include four isomers (*α*, *β*, *γ* and *δ*); each of them is defined on the basis of the number and localisation of the methyl groups on the phenol ring. Vitamin E (*α*-tocopherol) has a hydrophobic structure, and it is distributed in all membrane cells including plasmatic and mitochondrial membranes. It has been suggested that *α*-tocopherol is not randomly incorporated in the phospholipid bilayer, but it is segregated in specialised membrane complexes, like lipid rafts, where it is associated with PUFA present in phosphatidylcholine. The effect of this interaction is the decrease of the membrane cell fluidity and the increase of its rigidity. This event may change the activity of enzymes associated with lipid rafts in cell membranes^(99)^. Furthermore, this micronutrient represents the major lipid soluble chain-breaking antioxidant and it traps peroxyl-radicals and reactive oxygen species, which are produced during peroxidative reactions, by means of its chromanol ring^(100)^. Therefore, *α*-tocopherol modulates the action of free radicals and contributes to prevent the damage of cellular macromolecules end microrganelles, induced by the OS.

Overall, it may be hypothesised that these fat-soluble vitamins might directly or indirectly regulate the physical characteristic of the lipid rafts and modulate the fluidity plasmatic cell membranes, increasing the rigidity of these structures. A large series of the enzymes regulated by fat-soluble vitamins, such as tocopherol, are associated with lipid rafts and can change protein–lipid and protein–protein interactions and influence raft-embedded signal transduction pathways. These modifications may contribute to decrease the infective ability of the viruses, including SARS-CoV^(101)^.

### Modulation of immune response function

The modulation of immune response leading to the improvement of antiviral response derives the conceptual rationale for the inclusion of vitamins A, D and E in a possible multitherapeutic protocol for the treatment of patients with SARS-CoV-2-related infection.

These vitamins may contribute to improve normal immune response, by restoring the normal immune system activity, mainly by counteracting Th1/Th2/Th17 unbalance and modulating the amounts and the ratio among the pro-inflammatory and anti-inflammatory cytokines. As reported in the studies, vitamin D alone or in association with Tocilizumab is able to block the activity of IL-6 receptor and to promote the generation of Foxp3^+^ T-cells and to counteract IL-17 production. These cells modulate the immune response and contribute to turn off the production of pro-inflammatory cytokines. Furthermore, vitamin E also is able to prevent IL-6 release. A very recent report has shown that SARS-CoV-2 viral load (RNAemia) in serum is closely associated with drastically elevated IL-6 level in patients with severe disease (data not published). The combined use of fat-soluble vitamins might exert an even more beneficial effect in elderly patients, who are characterised by an impairment of immune system function. These individuals are characterised by a very high mortality in Italy during this epidemic outbreak (unpublished data)^(102–104)^.

Furthermore, these compounds present an additional anti-inflammatory activity mediated by the production of microRNA-122. These elements are short-cell RNAs which exert a wide series of regulatory cell activities and modulate also antiviral immune response.

According to Gu’s hypothesis, the immune system dysfunction is the most important cause of clinical deterioration and possible unfavourable outcome in the individuals with CoV disease^(14)^. Therefore, the possible usefulness of immune system restoration by using these fat-soluble vitamins might represent a crucial strategy with the purpose to prevent or to progressively inhibit the CRS. However, in their use with this indication, fat-soluble vitamins A, D, E should be considered not only as nutrients but also as real drugs with potential useful or dangerous effects. Unfortunately, to date, no studies have assessed the blood concentration of these fat-soluble vitamins in patients with SARS-CoV-2 as well as it is unknown whether deficiency in these micronutrients may be associated with a more severe course and outcome of this disease. Therefore, trials evaluating blood concentration of these compounds should be performed as soon as possible and the possible inclusion of fat-soluble vitamins in the treatment schedules of COVID-19 patients should be considered. However, the possible side effects of these compounds should be considered, and the dosage of blood fat-soluble vitamins should be provided. Based on all these pathogenic considerations, a possible protocol proposal for the treatment of patients with SARS-CoV-2 should consist of the following schedule already in the early phase of the disease:

(i) antiviral drugs to block viral replication and, mainly, the release of high amounts of viral proteins able to trigger a robust pro-inflammatory response;
(ii) immunomodulatory compounds with the purpose of restoring the unbalanced and dysregulated immune system function, including fat-soluble vitamins in association with Tocilizumab.

The early administration of these drugs could prevent the development of CRS with the subsequent clinical deterioration and deaths as well as it could decrease the need of intensive care beds.

On the basis of the available data concerning the dosage of fat-soluble vitamins as treatment of viral infections (HBV, HCV, HIV, etc.), it may be suggested that these micronutrients should be used as drugs and not as simple dietary supplements, with the purpose to obtain proper serum and tissue concentration^(78,79,103,105)^. To date, the possible effective dosage of these micronutrients for the therapy of the acute infection caused by SARS-CoV-2 is unknown, as no trials have been concluded in these patients with this purpose. Therefore, it may be conceivable to take into account the dose of vitamins A, D and E in the studies performed in patients with HBV/HCV/HIV persistent infection as well as in patients with autoimmune diseases, like rheumatoid arthritis^(103)^. The potential doses are indicated in Fig. 5^(77–79,105–114)^. In elderly people with moderate/severe deficiency in these micronutrients, it may be useful to consider schedules for the supplementation of all these vitamins with the purpose to reach normal tissue and serum concentrations of these fat-soluble vitamins. It may be hypothesised that this strategy, in the current absence of an effective vaccine against SARS-CoV-2, might improve the activity of immune system. This approach might both preventively attenuate the risk of the Th17-mediated pro-inflammatory response with potential deleterious effects and stimulate a regulatory T cell immune response leading to the prevention or to the reduction of ‘cytokine storm’ syndrome. In conclusion, in this paper, we have provided a rapid excursus on available data about a very life-threatening disease worldwide, known as SARS-CoV 2, then we have examined the crucial mechanisms potentially involved in the development of this severe illness. Since our research, we have identified the possible viral and host cell targets and suggested a rationale for an early poly-therapeutic approach. Unfortunately, several problems are also evident, including the dosage of antiviral drugs, of fat-soluble vitamins and Tocilizumab as well as the potential side effects of these treatments. Well-designed and well-sized protocols are needed to improve our knowledge in the immunopathogenesis of this complex disease, with the purpose to contribute to the control of this public health emergency.
